# *Arabidopsis thaliana* Leaf Epidermal Guard Cells: A Model for Studying Chloroplast Proliferation and Partitioning in Plants

**DOI:** 10.3389/fpls.2019.01403

**Published:** 2019-10-30

**Authors:** Makoto T. Fujiwara, Alvin Sanjaya, Ryuuichi D. Itoh

**Affiliations:** ^1^Department of Materials and Life Sciences, Faculty of Science and Technology, Sophia University, Tokyo, Japan; ^2^Department of Chemistry, Biology and Marine Science, Faculty of Science, University of the Ryukyus, Nishihara, Japan

**Keywords:** chloroplast, guard cell, plastid development, organelle inheritance, organelle partitioning, stoma

## Abstract

The existence of numerous chloroplasts in photosynthetic cells is a general feature of plants. Chloroplast biogenesis and inheritance involve two distinct mechanisms: proliferation of chloroplasts by binary fission and partitioning of chloroplasts into daughter cells during cell division. The mechanism of chloroplast number coordination in a given cell type is a fundamental question. Stomatal guard cells (GCs) in the plant shoot epidermis generally contain several to tens of chloroplasts per cell. Thus far, chloroplast number at the stomatal (GC pair) level has generally been used as a convenient marker for identifying hybrid species or estimating the ploidy level of a given plant tissue. Here, we report that *Arabidopsis thaliana* leaf GCs represent a useful system for investigating the unexploited aspects of chloroplast number control in plant cells. In contrast to a general notion based on analyses of leaf mesophyll chloroplasts, a small difference was detected in the GC chloroplast number among three *Arabidopsis* ecotypes (Columbia, Landsberg *erecta*, and Wassilewskija). Fluorescence microscopy often detected dividing GC chloroplasts with the FtsZ1 ring not only at the early stage of leaf expansion but also at the late stage. Compensatory chloroplast expansion, a phenomenon well documented in leaf mesophyll cells of chloroplast division mutants and transgenic plants, could take place between paired GCs in wild-type leaves. Furthermore, modest chloroplast number per GC as well as symmetric division of guard mother cells for GC formation suggests that *Arabidopsis* GCs would facilitate the analysis of chloroplast partitioning, based on chloroplast counting at the individual cell level.

## Introduction

Chloroplasts represent a structural feature of plant cells and support plant survival *via* their primary metabolism and high-level functions (Kirk and Tilney-Bassett, 1978; Mullet, 1988; López-Juez and Pyke, 2005). During plant vegetative growth, leaf cells contain a highly homogeneous population of chloroplasts with respect to size and shape. The number of chloroplasts per cell is achieved by binary fission of pre-existing organelles and partitioning into two daughter cells during cell division (Birky, 1983; Possingham and Lawrence, 1983). Thus, regulation of the chloroplast number in a given cell type is crucial for the cellular function and genetic inheritance of chloroplasts.

To investigate the nature of chloroplast number determination in plant cells, leaf mesophyll cells of representative species have played a major role [*e.g.*, Boasson and Laetsch, 1969 (for tobacco, *Nicotiana tabacum*); Honda et al., 1971 (for spinach, *Spinacia oleracea*); Boffey et al., 1979 (for wheat, *Triticum aestivum*); Lamppa et al., 1980 (for pea, *Pisum sativum*); and Pyke and Leech, 1991 (for *Arabidopsis thaliana*)]. These cells are physiologically important for photosynthesis and show a high degree of structural and functional homogeneity. Early systematic observation analyses of isolated tissues and cells (Boasson and Laetsch, 1969; Possingham and Saurer, 1969; Boffey et al., 1979; Lamppa et al., 1980; Thomas and Rose, 1983; Pyke and Leech, 1991) provided much useful information on chloroplast number determination, including the notion that chloroplasts (plastids) are not synthesized *de novo* but replicate by division and the observation that leaf mesophyll chloroplast number is sensitive to various environmental and plant-endogenous factors. With respect to the latter, in spinach, light has a positive impact on chloroplast division during leaf disc culture compared with dim or dark conditions (Possingham and Lawrence, 1983). In the first leaves of wheat, cell volume is positively correlated with chloroplast proliferation (Ellis and Leech, 1985; Pyke and Leech, 1987). In *Arabidopsis*, the genetic background affects chloroplast proliferation; the average chloroplast number per cell in first leaves is 121 in the Landsberg *erecta* (L*er*) ecotype and 83 in the Wassilewskija (Ws) ecotype (Pyke and Leech, 1994; Pyke et al., 1994). Leaf mesophyll cells have also contributed to understanding the genetic control of chloroplast division; for instance, screening mutants impaired in chloroplast proliferation and characterizing gene functions involved in chloroplast division have revealed over 20 genes encoding chloroplast division machinery components or chloroplast regulatory factors (Gao and Gao, 2011; Miyagishima et al., 2011; Basak and Møller, 2013; Osteryoung and Pyke, 2014; Li et al., 2017).

By contrast, studies on the replication of chloroplasts in non-mesophyll cells (*e.g.*, pavement cells in leaf epidermis; Itoh et al., 2018) are scarce. Recently, the regulation of chloroplast division has been reported to differ between leaf tissues (Fujiwara et al., 2018; Itoh et al., 2018), although the detailed mechanism remains unknown. Additionally, while the analyses of suspension-cultured BY-2 cells and leaf mesophyll protoplasts in tobacco and shoot apical meristem and leaf primordial cells in *Arabidopsis* (Nebenführ et al., 2000; Sheahan et al., 2004; Seguí-Simarro and Staehelin, 2009) have provided major insights, how chloroplast (plastid) partitioning is regulated in plants is still unclear. Thus, despite considerable effort, fundamental questions in chloroplast research remain, such as (i) how is chloroplast number per cell coordinated in plant tissues and (ii) how is chloroplast partitioning regulated at cell division.

## History of Research on Guard Cell Chloroplast Number

**Figure 1:**
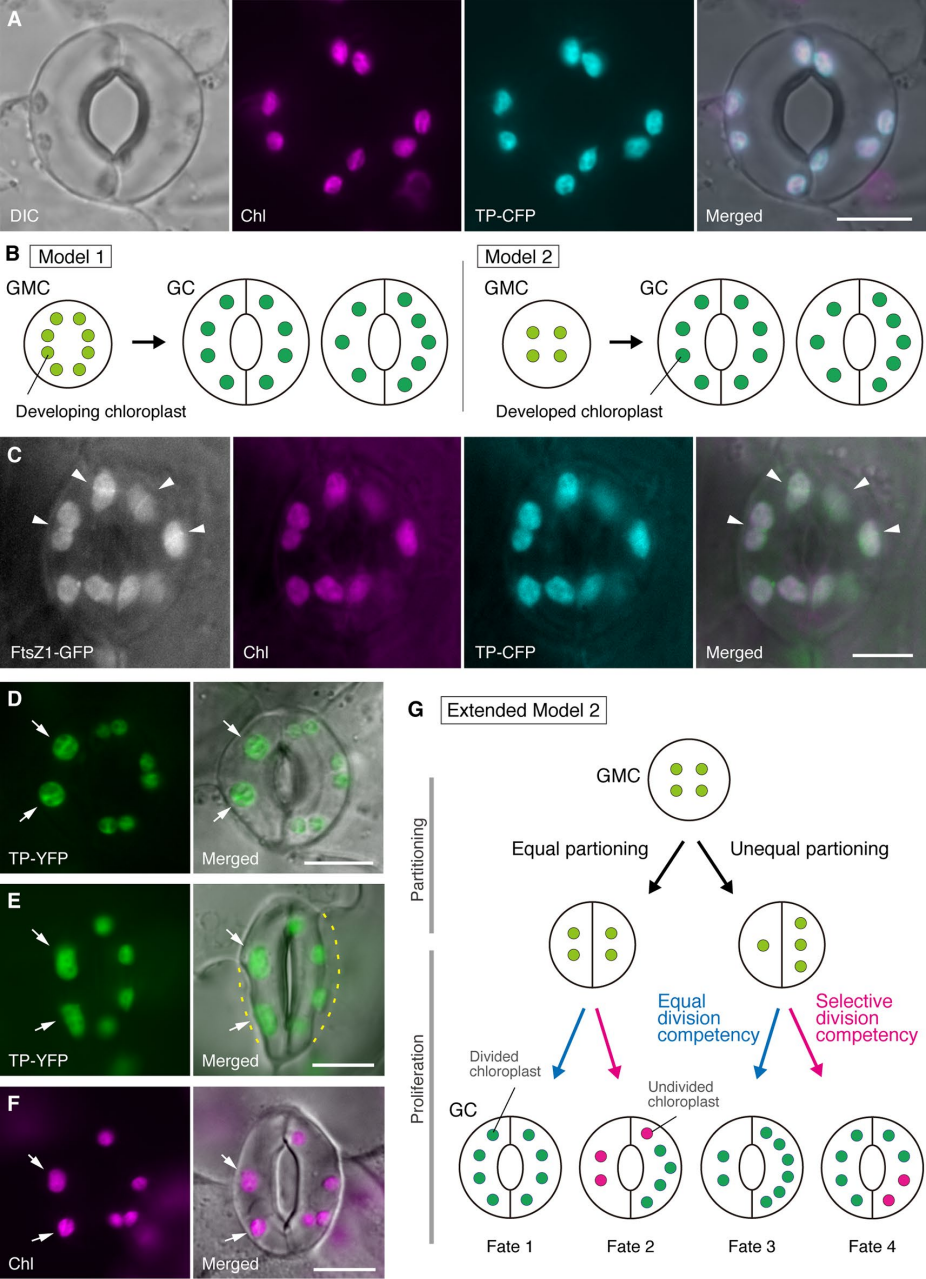
Simplified models and microscopy evidence for the control of chloroplast number in stomatal guard cells (GCs). **(A)** A typical stoma (GC pair) in abaxial epidermis of the *Arabidopsis* leaf blade expressing a stoma-targeted fusion of the transit peptide (TP) with cyan fluorescent protein (CFP; TP-CFP). **(B)** Two models of chloroplast number determination in GCs, involving either chloroplast partitioning (model 1) or both chloroplast proliferation and partitioning (model 2) during GC development from guard mother cells (GMCs). **(C)** A GC pair in adaxial epidermis of *Arabidopsis* leaf petiole expressing TP-CFP and FtsZ1 fused to the green fluorescent protein (GFP; FtsZ1-GFP). **(D**–**F)** GC pairs in abaxial epidermis of *Arabidopsis* leaf blade with **(D**, **E)** or without **(F)** the expression of TP fused to the yellow fluorescent protein (YFP; TP-YFP). **(F)** Chlorophyll autofluorescence (Chl) was used as a chloroplast marker. **(G)** Extended model 2, representing the involvement of equal and unequal chloroplast partitioning following GMC division and subsequent division of GC chloroplasts with equal (blue line) or selective (red line) division competency, which would result in four types of chloroplast number determination (Fates 1–4) during late stomatal development of *Arabidopsis* leaves. **(A**, **C**–**F)** Epifluorescence microscopy was performed with an Olympus IX71 inverted microscope using plant materials as previously described (Fujiwara et al., 2017, Fujiwara et al., 2018). Fluorescence signals of chlorophyll, CFP, GFP, and YFP are pseudo-colored in magenta, cyan, green (in merged image only), and green, respectively. Indications in panels are as follows: arrowhead, the FtsZ1 ring; arrow, enlarged GC chloroplast; dashed line, cell shape. Scale bar = 10 µm.

Stomatal GCs in the shoot epidermis generally contain chloroplasts and control gas exchange between the leaf mesophyll and the atmosphere (Sachs, 1875; Taiz et al., 2015; see **Figure 1A**). The first investigation of GC chloroplast number in leaves was performed over a century ago in naturally grown *Drosera* plants (Macfarlane, 1898). This study demonstrated that, like other plant and cell structural features, GC chloroplast number per cell in a putative hybrid derived from a cross between *Drosera filiformis* and *Drosera intermedia* was intermediate between the two species, implying that GC chloroplast number could be used to determine the genetic makeup of a plant. Important observations were subsequently reported on the differences in GC chloroplast number among plant species (Sakisaka, 1929) and the relatively stable chloroplast number in GCs in the leaf epidermis of mulberry (*Morus* spp.; Hamada and Baba, 1930) and in mature leaves of several *Brassica* species (Iura, 1934). Furthermore, analysis of autopolyploid sugar beet (*Beta vulgaris*) plants revealed that the GC chloroplast number in leaves is positively correlated with the nuclear ploidy level of plants (Mochizuki and Sueoka, 1955). More in-depth and comprehensive analyses were then conducted using various plant samples to investigate the relationship of chloroplast number and stomatal size with the ploidy level (*e.g.*, Frandsen, 1968). In these analyses, chloroplast counting at the stomatal (GC pair) level was frequently adopted, which excluded the effect of biased chloroplast distribution between paired GCs (*e.g.*, Mochizuki and Sueoka, 1955; Frandsen, 1968), revealing that the average GC chloroplast number in leaves or cotyledons in approximately 80 species, variants, or hybrids ranged from 2.8 to 40.0 in diploids (2×) and 5.0 to 73.5 in tetraploids (4×). In addition, whole-genome duplication events in plants (*i.e.*, 1× to 2×, 2× to 4×, etc.) caused an approximately 1.7-fold increase in GC chloroplast number with high fidelity (reviewed in Butterfass, 1973). These results encouraged investigations into ploidy level in various tissues and plants obtained *via* tissue culture, crossing, or natural cultivation, in combination with chemical (*e.g.*, colchicine) or radiation treatments (*e.g.*, Jacobs and Yoder, 1989; Singsit and Veilleux, 1991; Qin and Rotino, 1995). While GC chloroplast number has been studied in stomatal biology (Lawson, 2009) and cytology to understand chloroplast multiplication (Butterfass, 1979; see below), it has largely served as a reliable and convenient marker for the detection of hybrids, species, and variants and for the estimation of ploidy levels of target plant tissues.

## Utility of Leaf Guard Cells For the Analysis Of Chloroplast Number Control

Leaf mesophyll cells have long been employed as a primary model for the analysis of chloroplast number. While they have advantages for the study of the effects of environmental conditions on chloroplast division (*e.g.*, the light-cytokinin signaling; Boasson and Laetsch, 1969; Possingham and Lawrence, 1983; Okazaki et al., 2009; Chiang et al., 2012), they are limited in some respects. Firstly, leaf mesophyll cells vary in size and shape and are distributed deep within the leaf, which makes it difficult to manipulate intact tissues. Secondly, the susceptibility of leaf mesophyll chloroplast proliferation to environmental stress and plant growth conditions can prevent reliable comparisons between studies. The leaf mesophyll chloroplast number per cell in *Arabidopsis* Columbia (Col) ecotype has been reported variously as 76 (Kinsman and Pyke, 1998), 80–100 (Stokes et al., 2000), 70 (Tirlapur and König, 2001), 41 (Yoder et al., 2007), 40–60 (Okazaki et al., 2009), and 30–40 (Kawade et al., 2013). Furthermore, it is almost impossible to assess the contribution of chloroplast partitioning to final chloroplast number per mesophyll cell during leaf development, although this is thought to be determined by the balance between the rate of cell division and rate of chloroplast division. To uncover the mechanism of chloroplast number control in vegetative leaf cells, a model system that overcomes the above issues is required.

Stomatal GCs (see **Figure 1A**) exhibit the characteristics of a model system for understanding the mechanism of chloroplast number control. GCs are highly uniform in size and shape within a tissue, and their scattered but dense distribution in the outermost layer of shoots facilitates their detection by light and fluorescence microscopy. GCs are also derived from protodermal cells in the shoot apical meristem or from embryonic epidermal cells, and their developmental sequence through meristemoids (a stomatal precursor with meristematic activity) and guard mother cells (GMCs; a precursor of GC pairs) is established in detail (Zhao and Sack, 1999; Nadeau and Sack, 2002; Kalve et al., 2014). Late stomatal development involves a single round of symmetric GMC division, which enables the assessment of chloroplast distribution and partitioning before and after cytokinesis. From the perspective of practical experiments, leaf GCs are suitable for microscopy. It was previously shown that chloroplast number per cell in leaf GCs of *Sinapis alba* was less affected by different light conditions than that in leaf mesophyll cells (Wild and Wolf, 1980). Additionally, the difference in GC chloroplast number in leaf petioles is relatively minor among the three *Arabidopsis* ecotypes Col, L*er*, and Ws (Fujiwara et al., 2018). Furthermore, endoreduplication, which impacts the development of leaf mesophyll, pavement, and trichome cells, has not been detected in *Arabidopsis* leaf GCs (Melaragno et al., 1993), which would assure the interpretations of chloroplast number data at the 2C level of cells. Together, these reports suggest that leaf GCs are potentially an excellent model for the systematic analysis of chloroplast number dynamics in a particular cell lineage.

## *Arabidopsis* Leaf Guard Cells as a Model for Studying the Control of Chloroplast Number

In the history of GC chloroplast research, chloroplast counting at the stomatal (GC pair) level has served an equally important role in determining the chloroplast number as counting at the individual GC level. Both methods produce the same mean chloroplast number (Butterfass, 1973). When the variation in chloroplast distribution in paired GCs and its underlying mechanism is a subject of focus, detailed information of chloroplasts at the individual cell level, *i.e.*, their size, shape, and intracellular localization, is essential. Chloroplast (plastid) proliferation during the GMC–GC differentiation was previously investigated in several plant species (Butterfass, 1973, Butterfass 1979). These studies proposed two models for determining the terminal chloroplast number in GCs in different plant species (**Figure 1B**): one (model 1; sugar beet) involves only chloroplast partitioning at GMC division, and the other [model 2; alsike clover (*Trifolium hybridum*)] involves not only chloroplast partitioning but also chloroplast proliferation during GC development.

In the era of molecular genetics, genomics, cell imaging, and other interdisciplinary analyses, there are many possibilities for the further characterization of the chloroplast partitioning mechanism. *Arabidopsis* leaf GCs may be one of the best model systems for this purpose. Several studies have examined the GC chloroplast number in the leaves or cotyledons of *Arabidopsis* (Hoffmann, 1968; Pyke and Leech, 1994; Pyke et al., 1994; Robertson et al., 1995; Keech et al., 2007; Chen et al., 2009; Yu et al., 2009; Higaki et al., 2012; Fujiwara et al., 2018). These GCs exhibit a modest number of chloroplasts, ranging from 3.5 to 5.5 on average. To date, no studies have examined the alterations in chloroplast (plastid) number during stomatal development. However, microscopic evidence from stomatal development analyses (*e.g.*, Zhao and Sack, 1999; Hachez et al., 2011) and our preliminary observations indicate that GMCs may contain smaller numbers of developing chloroplasts than GCs and that chloroplast proliferation may occur during GC differentiation. To test this, the formation of the chloroplast division machinery in GCs was monitored with the probe FtsZ1 fused to the green fluorescent protein (FtsZ1-GFP) (Fujiwara et al., 2008). A transgenic line, simultaneously expressing a transit peptide (TP)-fused CFP and FtsZ1-GFP to visualize the stroma and FtsZ1 ring, respectively, was examined by epifluorescence microscopy (Fujiwara et al., 2017). Expanding leaf petioles (fifth leaves of 4-week-old seedlings) were employed. As a result, GCs with symmetrically constricting chloroplasts were detected (**Figure 1C**). These chloroplasts formed the FtsZ1 ring, a chloroplast division ring on the stromal surface of the inner envelope membrane in leaf mesophyll and pavement cells (Vitha et al., 2001; Fujiwara et al., 2008), at the equatorial constriction site. Consistent with the stomatal patterning in *Arabidopsis* leaf development (Donnelly et al., 1999; Andriankaja et al., 2012), dividing chloroplasts were detected at the late, as well as early, stage of leaf expansion. Thus, model 2 is most likely the best fit for *Arabidopsis* leaf GCs.

## Relationship Between Chloroplast Proliferation And Expansion in *Arabidopsis* Leaf Guard Cells

Furthermore, an unexpected phenotype of GC chloroplast morphogenesis was observed in mature GCs (**Figures 1D, E**). When epidermal peels of fully expanded leaves (third–fourth leaf blades of 4-week-old seedlings) from a TP-fused yellow fluorescent protein (YFP) line were microscopically characterized (FL6-5 line; Fujiwara et al., 2018), some stomata showed unequal chloroplast distribution patterns in GC pairs, while most leaf stomata showed equal or similar chloroplast distribution patterns (Robertson et al., 1995; Fujiwara et al., 2018). Within the GC pair of a stoma, the size of chloroplasts in the GC containing smaller numbers of chloroplasts was larger than in the other GCs in the pair containing larger numbers of chloroplasts (**Figures 1D, E**). In this way, GCs probably maintain the total chloroplast volume per cell at a constant level during cell growth. Enlarged chloroplasts represented the terminal phenotype and could no longer divide in expanded leaves. These results were confirmed in several independent experiments, irrespective of the expression of a TP-fused fluorescent protein for stroma labeling (**Figure 1F**).

This GC chloroplast phenotype is interpreted as a compensation mechanism for chloroplast expansion, which was well documented in leaf mesophyll cells defective in the control of chloroplast division (Pyke and Leech, 1994; Pyke et al., 1994). To date, only one study (Ellis and Leech, 1985) has reported a negative correlation between chloroplast number and chloroplast size in leaf mesophyll cells of wheat, whereas many studies have reported a positive correlation between cell volume and chloroplast number in normal leaf mesophyll cells (Leech and Pyke, 1988; Pyke, 1997). Whereas imbalances in GC chloroplast number occur at low frequency (Fujiwara et al., 2018), chloroplast heterogeneity in GC pairs indicates that unequal chloroplast partitioning could trigger differential chloroplast growth between wild-type leaf cells in *Arabidopsis*, despite symmetric cell division.

The chloroplast compensation effect in GCs may be less strict than in leaf mesophyll cells. GCs might be able to withstand scarcity or complete loss of total chloroplast volume per cell in severely impaired chloroplast division mutants, such as in *Arabidopsis arc6* and *atminE1* and tomato *suffulta*, whereas many mutant GCs showed reduced chloroplast number and enlarged chloroplast size similarly to the leaf mesophyll cells (Robertson et al., 1995; Forth and Pyke, 2006; Chen et al., 2009; Fujiwara et al., 2018). In a late chloroplast division mutant, *arc5*, the reduction in GC chloroplast number was not associated with a significant increase in chloroplast size, unlike in leaf mesophyll cells (Pyke and Leech, 1994). A lower degree of chloroplast expansion in GCs than in mesophyll cells (Pyke and Leech, 1994; Barton et al., 2016), and the variation in chloroplast expansion among GCs, might underlie such a wide permissible range of total chloroplast volume per GC. Furthermore, the timing of chloroplast division during GMC–GC differentiation might significantly affect the terminal GC chloroplast phenotype. Although further detailed characterization is required to address this issue, it seems plausible that *Arabidopsis* leaf GCs represent a system to investigate the unexploited aspects of chloroplast number control in plant cells.

## A Working Model For Chloroplast Number Determination in *Arabidopsis* Leaf Guard Cells

On the basis of the above, we propose a working model (an extended model 2) for the analysis of chloroplast number in GCs (**Figure 1G**). The final chloroplast number per GC is determined by chloroplast partitioning at GMC division and chloroplast proliferation in GCs. During GMC division, chloroplasts may undergo either equal or unequal partitioning. During chloroplast proliferation, GC chloroplasts will proliferate with either equal (blue line) or selective (magenta line) division competency. For example, if equally partitioned chloroplasts possess equivalent division competency, equal chloroplast numbers will occur in the GC pair (Fate 1). If unequally partitioned chloroplasts possess equivalent division competency, chloroplasts will increase at the same rate within the GC pair (Fate 3). If selective chloroplast division occurs in GCs, the balance of chloroplast number in the GC pair will change after GMC division (Fates 2 and 4). It is currently difficult to find support for “selective chloroplast division,” but if Fates 1 and 4 actually predominate in GCs, then they might possess a mechanism that controls total chloroplast volume per cell, as in leaf mesophyll cells. The model raises two issues: (i) Are GC chloroplasts properly partitioned into daughter cells and how do they partition? And (ii) is division competency of GC chloroplasts coordinately regulated?

Regarding issue (i), whether chloroplast inheritance occurs by random distribution of multiple chloroplasts in the cytoplasm or by positive chloroplast partitioning mechanism(s) has been a long-standing concern (Butterfass, 1969; Birky, 1983; Hennis and Birky, 1984; Nebenführ, 2007; Sheahan et al., 2016). Intriguingly, in *Arabidopsis arc6*, leaf or cotyledon GCs have zero to three chloroplasts, and in chloroplast-deficient GCs, non-photosynthetic plastids still exist in vesicular to elongated forms (Robertson et al., 1995; Chen et al., 2009; Fujiwara et al., 2018). No GCs devoid of plastids *per se* have been found in *arc6*, and no explanation for this has been forthcoming, despite the disruption of the chloroplast division apparatus (Vitha et al., 2003). Accordingly, it will be important to examine the replication and morphology of *arc6* chloroplasts in stomatal lineage studies. *Arabidopsis* mutant research may also give another clue for this issue. The observation that 18% of cotyledon GCs in the *crumpled leaf* (*crl*) mutant contain no plastidic structures in the cytoplasm, while 100% of the leaf mesophyll cells contain one to four enlarged chloroplasts (Asano et al., 2004; Chen et al., 2009), is of great importance. CRL is a chloroplast outer-envelope protein with an unknown function. Understanding CRL protein function may provide insights into the mechanism(s) of chloroplast partitioning. The analysis of chloroplast proliferation and partitioning in leaf mesophyll cells in *Arabidopsis arc* mutants and other transgenic lines has promoted research into the proliferation and partitioning of non-mesophyll plastids. Likewise, results obtained in GCs may be transferrable to other cell systems.

## Final Remark

The GC model opens many prospects for the development of chloroplast biology. For example, while cytoskeletal systems are known to regulate chloroplast morphology, movement, and partitioning (Sheahan et al., 2016; Wada, 2016; Erickson and Schattat, 2018), the role of each regulatory gene in chloroplast proliferation and partitioning in plants has received little attention. On the other hand, once it becomes possible to impair GC chloroplast number or morphology *via* various experimental strategies, new insights into the molecular control of chloroplast morphogenesis in stomatal lineage cells may be provided. Additionally, in conjunction with quantitative analyses of chloroplast number during stomatal development, mathematical modeling may offer a new avenue for these investigations. This paper presents current knowledge of how GC chloroplast number is controlled and highlights the potential usefulness of *Arabidopsis* leaf GCs for understanding chloroplast proliferation and partitioning.

## Data Availability Statement

All datasets for this study are included in the article/ supplementary material.

## Author Contributions

MF conceived the study and wrote the manuscript. MF and AS conducted the experiments. AS and RI conducted the analyses. All authors read and approved the final manuscript.

## Funding

This work was supported by the Ministry of Education, Culture, Science and Technology of Japan under KAKENHI (grant nos. 19K05831 to MF and 18K06314 to RI).

## Conflict of Interest

The authors declare that the research was conducted in the absence of any commercial or financial relationships that could be construed as a potential conflict of interest.
